# Prevalence of Residential Dampness and Mold Exposure in a University Student Population

**DOI:** 10.3390/ijerph13020194

**Published:** 2016-02-05

**Authors:** Mathieu Lanthier-Veilleux, Mélissa Généreux, Geneviève Baron

**Affiliations:** 1 Department of Community Health Sciences, Faculty of Medicine and Health Sciences, Université de Sherbrooke, Sherbrooke, QC J1H 5N4, Canada; mgenereux.agence05@ssss.gouv.qc.ca (M.G.); gbaron.agence05@ssss.gouv.qc.ca (G.B.); 2 Eastern Township's Public Health Department, 300, King Est street, Sherbrooke, QC J1G 1B1, Canada

**Keywords:** dampness, mold, housing, student population

## Abstract

The impact of residential dampness or mold on respiratory health is well established but few studies have focused on university students. This study aims to: (a) describe the prevalence of exposure to residential dampness or mold in university students according to socio-geographic factors and (b) identify associated housing characteristics. A web survey was conducted in 2014 among the 26,676 students registered at the Université de Sherbrooke (QC, Canada). Residential dampness and mold being closely intertwined, they were considered as a single exposure and assessed using a validated questionnaire. Exposure was compared according to socio-geographic and housing characteristics using chi-square tests and logistic regressions. Among the 2097 participants included in the study (response rate: 8.1%), over 80% were tenants. Residential exposure to dampness or mold was frequent (36.0%, 95% CI: 33.9–38.1). Marked differences for this exposure were noted according to home ownership (39.7% *vs.* 25.5% among tenants and owners respectively; OR = 1.92%, 95% CI: 1.54–2.38). Campus affiliation, household composition and the number of residents per building were associated with exposure to dampness or mold (*p <* 0.01), while sex and age were not. Exposure was also associated with older buildings, and buildings in need of renovations and lacking proper ventilation (*p* < 0.001). This study highlights the potential risk of university students suffering from mold-related health effects given their frequent exposure to this agent. Further research is needed to fully evaluate the mold-related health impact in this at risk group.

## 1. Introduction

The literature has reported extensively on the role of the indoor environment as a crucial health determinant [1]. Living with indoor contaminants, such as excessive house dust mite, fine particulate matter and formaldehyde emissions, tobacco toxic compounds, various mold species and other volatile organic compounds, is known to have significant respiratory health effects [1,2,3]. Health impact studies on exposure to mold are challenging because mold is ubiquitous yet often not visible, with a wide variety of species causing multiple health outcomes [1,2,4]. Indeed, mold species can trigger hypersensitivity reactions in atopic individuals [4], but their toxicity and inflammatory characteristics can also cause irritative symptoms in both atopic and non-atopic patients [1]. In all, residential dampness or mold are known to contribute to the development, or exacerbation, of atopic diseases such as asthma [1,5,6,7] and allergic rhinitis [1,8], as well as respiratory infections [1,9,10].

Most studies interested in residential dampness or mold have focused on children or a general adult population [1,6]. University students are typically considered young and healthy [11] but also share socioeconomic characteristics typical of a vulnerable population, such as being tenants and having a low income [12]. Moreover, housing instability, which leads to frequent tenant turnover, could result in carelessness in housing maintenance and upkeep by both landlords and occupants. This situation may contribute to residential dampness or mold persistence over time in rental units [13,14,15], but few studies have confirmed this hypothesis. Some university students are likely more at risk than others. For example, socioeconomically disadvantaged students may show fewer skills at resolving housing issues with their landlord than students with higher socioeconomic status or with those owning their housing [13,16]. Previous studies have also identified specific socio-demographic factors such as socioeconomic status [17], race [17] and home ownership [13] as factors associated with exposure to residential dampness or mold.

The geographic distribution of such exposure is also important to assess as the prevalence of residential dampness or mold differs greatly between countries. This prevalence has been estimated to be at least 20% in many Western countries [18], and, more specifically, 24% in the United States [19] and between 14% and 38% in Canada [20]. While climate could explain some of these differences [21], it cannot explain neighborhood differences seen within the temperate city of Montreal, which ranged from 30% to 52% in a recent study [22].

Socio-geographic factors alone do not fully characterize a population’s vulnerability to exposure to residential dampness or mold. Housing characteristics associated with dampness or mold may therefore serve the purpose of better targeting at-risk population for preventive and corrective interventions. Such at-risk buildings are commonly old, lack proper ventilation, need renovations, have carpeted floors and a peripheral heating system [13,23]. Occupant behavior in terms of maintaining proper ventilation and heating may also have an important impact on air quality [24]. In fact, specific housing investments allowing better heating, proper ventilation, and regular maintenance already have been associated with important health improvements [24,25].

This study aims to: (1) describe the prevalence of exposure to residential dampness or mold in university student housing according to socio-geographic factors and (2) determine associated housing characteristics.

## 2. Experimental Section

### 2.1. Setting

The standard minimal age to attend university in Quebec is 18 years old. Most Quebec universities, including the Université de Sherbrooke, are publicly funded. Student tuition—approximately CAD1500 per semester—is considered low by international standards [26]. Moreover, government grants and loans are offered to students with a low socioeconomic status. Many universities own accommodations, but available units are limited and most students live in privately-owned rental units. In Quebec, 38.6% of housings were rental units in 2012 [27]. Their 3.0% vacancy rate was near the Canadian rate of 2.8% [28], but well below the USA average of 7.3% [29]. Many students move away from their families to attend university. Therefore, many students go back to their families during weekends and holidays. Another particularity worth mentioning is that all electricity production in Quebec has been nationalized since in the 1960s and therefore electrical heating or ventilation costs are much lower than in most other northern countries.

**Figure 1:**
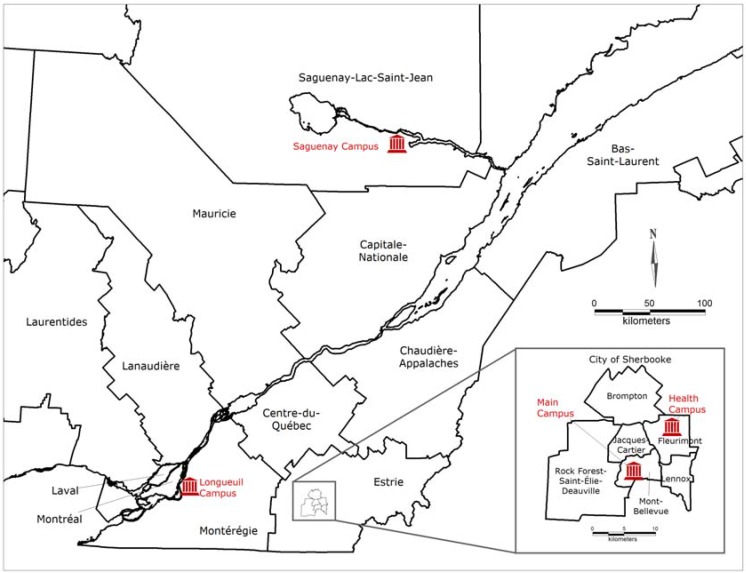
Geographic distribution of the Université de Sherbrooke campuses. Map of Quebec administrative regions with a zoom on the city of Sherbrooke; campuses are indicated by red symbols.

The Université de Sherbrooke (Quebec, Canada) is a French-speaking university with multiple campuses attended by over 20,000 students each year. As shown in Figure 1, its campuses are geographically distributed throughout the province of Quebec. The University’s main and health campuses are located in two districts of Sherbrooke, a central city in the Eastern Townships region with 150,000 inhabitants. A third campus is located in Longueuil (405,000 inhabitants), a city roughly one-tenth the size of the neighboring metropolitan area of Montreal. A fourth campus is located further north, in a mid-sized city named Saguenay (145,000 inhabitants). Moreover, roughly one-fifth of students are registered for distance studies throughout the province of Quebec.

### 2.2. Recruitment

The study population consists of all students registered at the Université de Sherbrooke during the 2014 winter trimester. Each of the 26,676 students was contacted via their university email in early March 2014 and two follow-up emails were sent each subsequent week. The expected response rate is generally lower for e-mail surveys (around 21% [30]) than for mail surveys [31]. Nevertheless, university students are more easily reached this way as all students regularly use their Université de Sherbrooke email account. While web surveys could lead to a selection bias in a general population (as a low socioeconomic status usually indicates lower internet access), this may not be the case for university students. To maximize response rate, many strategies proposed by Dillman [32,33] were applied such as the use of reminders, support from known organizations and incentives (twenty CAD50 gift certificates for the university shop). A web link allowed students to access the consent form and the online survey. Only adults (18 years and older) understanding French and with a primary address in Quebec were included in the study. Students registered at University of the Third Age were excluded, as they were not expected to share the same vulnerability characteristics as the rest of the student population. Institutional and organizational approvals were obtained from the Centre de Recherche du CHUS research ethic board, from the university and from the student federations (FEUS and REMDUS) before contacting students. The project identification code for CHUS ethic board approval is 2014-752, 14-022. Each respondent had to sign a consent form electronically before completing the questionnaire.

### 2.3. Response Rate

Of the 26,676 students in the study population, 3029 (11.4%) students were recruited via their university email. However, 726 (2.7%) did not consent to the study and 75 (0.3%) were not eligible, mostly because their primary housing was located outside of the province of Quebec. A further 131 respondents (0.5%) were excluded because they did not respond to key sections of the questionnaire such as questions on dampness or mold exposure. Overall, 2097 questionnaires were included in the analysis, which corresponds to a 7.9% response rate but constitutes 69.2% of the 3029 recruited students. When considering our target population (*n* = 26,676) and after correction for ineligibility among recruited students (2.5%), the final participation rate was 8.1%. Consequences of this low participation rate are discussed further below in the limitations section.

### 2.4. Questionnaire Development

For validity and comparison purposes, the survey questionnaire was developed using questions selected from validated questionnaires. The chosen criteria for any sign of dampness or mold exposure have a sensitivity and specificity of 75% and 71% respectively, when considering an evaluation by a hygienist as a gold standard [34]. The study questionnaire was mostly based on the French versions of the European Community Respiratory Health Survey I and II (ECRHS), the Montreal Children respiratory health study and the Canadian collectivity Health Survey [22,35,36,37]. Exact wording of questions from these surveys was used whenever possible. Moreover, two local scholars with appropriate expertise in the area of environmental health and questionnaire development independently assessed content validity of the survey by reviewing each question. The web version was developed using the Limesurvey® platform and hosted on the Université de Sherbrooke server. A pilot test of the online survey was conducted with six students in order to assess completion time and comprehension. The final version of the online survey included up to 144 questions with numerous conditional sections and took 10 to 20 min to complete. To facilitate comprehension and reduce completion time, priority was given to multiple short questions rather than fewer, but more complex, questions. Dichotomous or multiple choice questions were thus used whenever possible. Finally, other topics covered in this survey included respiratory health (asthma-like symptoms, allergic rhinitis and respiratory infections). The extent to which respiratory diseases among students were associated to residential mold or dampness will be addressed in a subsequent paper.

### 2.5. Conceptual Model

**Figure 2:**
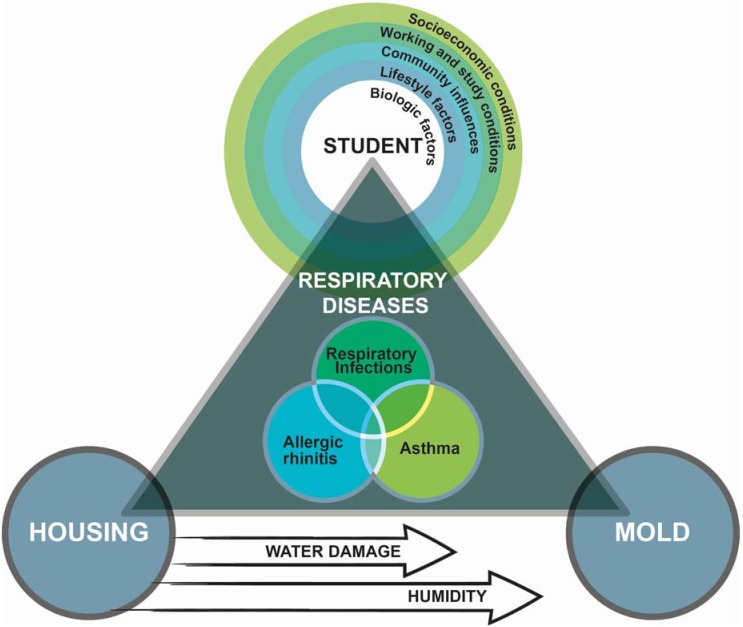
Conceptual model illustrating the impact of mold exposure in student housing on respiratory diseases.

To better represent associations and interactions between exposure to dampness or mold and respiratory diseases, we developed a conceptual model (presented in Figure 2) based on the epidemiologic triangle [38] and the ecologic model (adapted from Dhalgren) [39]. Each peak of the triangle corresponds to the host (*i.e*., student), the environment (*i.e*., housing) and the agent (*i.e*., mold). Host characteristics that may influence the relationship between the environment and the agent are depicted using the ecologic model with concentric circles representing various levels of influence. The largest circle, socioeconomic conditions, is particularly important since it is closely related to numerous housing characteristics, such as housing quality, maintenance and overcrowding, which contribute to indoor air quality and health risks. At the center of our conceptual model, the three key respiratory diseases (asthma, allergic rhinitis and respiratory infections) overlap (adapted from Hong’s Venn *et al.*,) [40] to illustrate the clinical interactions between these diseases and the fact that they are often simultaneously present in vulnerable patients. In addition, two arrows at the bottom of the conceptual model highlight the factors, such as dampness or water damage, which are important contributors to mold growth and persistence.

This conceptual model represents some of the interactions between housing, students and mold. To simplify the model, many other contributors to respiratory health, such as house dust mite, smoking, wood fire and pets, have been omitted.

### 2.6. Variables

To evaluate student exposure to residential dampness or mold, respondents were asked for signs in the past twelve months at their term-time address of: (a) visible mold; (b) mold odor; (c) excessive humidity such as wet or damp spots on surfaces; and (d) present or past water leaks [22,34] which were not cleaned within 48 h (as per EPA recommendations [41]). Residential dampness and mold being closely intertwined, any one of these four signs was regarded as a potential mold problem, as described in the literature [6,9,34,42]. Respondents were characterized according to the following socio-geographic factors: (1) demographic factors such as sex, age, spoken language at home, country of birth and residential location during their term (region, city and district); (2) academic characteristics such as campus, full-time *vs.* part time studies and grade level and; (3) socioeconomic factors such as home ownership, household composition, annual family income and housing expenses. Annual family income included the student’s and, when relevant, spouse’s income, as well as the income from any other sources (e.g., family allowance). The six-digit postal code corresponding to the term-time address was geocoded to identify the corresponding region, city and district (6% missing data). Housing characteristics were evaluated, including building type and construction year, the number of occupants per residence, ventilation and heating systems, the need for renovation as well as the presence of: carpeting, rodents, a dehumidifier, a humidifier and a thermostat.

### 2.7. Statistical Analysis

Frequency distributions and crosstables were used to describe the study sample. Only prevalence of residential dampness or mold exposure with a coefficient of variation below 15% are presented and those exposures were analyzed according to socio-geographic and housing characteristics. Chi-square (χ [2]) tests and univariate regressions were used to measure crude associations with residential exposure to mold or dampness. Univariate rather than multivariate logistic regression was used to calculate odds ratios (OR) and 95% confidence intervals (95% CI) because this study aims to describe factors associated with exposure to residential mold or dampness, rather than establish a causal relationship. An alpha value of 0.05 was used for statistical significance. Results were weighted for sex, age and campus affiliation using publicly available data. Thus, each stratum of respondents (based on sex, age group and campus affiliation) was given a weight. This weight was obtained by dividing the proportion of this stratum in the study population by the corresponding proportion in the sample. All analyses were performed using SPSS version 21.

## 3. Results

### 3.1. Socio-Demographic Characteristics

Socio-demographic characteristics are presented in Table 1. Most students were born in Canada, spoke French at home and studied full time. Over half of respondents were young students, baccalaureate candidates and studied at the main campus. More than 80% of respondents were tenants and more than half declared a family annual income below CAN25,000. A comparison of unweighted and weighted data shows that women, younger students, those studying full time and those studying at the main or health campuses (both located in Sherbrooke) were over-represented in the sample.

**Table 1:** Socio-demographic characteristics of respondents (*n* = 2097), Université de Sherbrooke students (QC, Canada), winter 2014.

Characteristic	Group	Unweight % (n)	Weighted * % (n)
Sex	Women	70.3% (1391)	56.3% (1110)
Country of birth	Canada	87.3% (1728)	84.6% (1667)
Spoken language at home	French spoken at home	97.5% (1928)	96.6% (1903)
Mode of study	Full time	89.7% (1767)	79.2% (1556)
Age	18 to 23 years old	55.3% (1094)	40.1% (790)
	24 to 30 years old	31.5% (623)	29.1% (573)
	31 years old and older	13.3% (263)	30.9% (609)
Grade	Baccalaureates	61.9% (1215)	51.8% (1017)
	Masters	26.7% (525)	35.6% (700)
	Doctorates or post-doctorates	11.4% (224)	12.6% (248)
Campus affiliation	Main campus	65.5% (1291)	54.1% (1067)
	Health campus	17.7% (349)	10.7% (210)
	Longueuil campus	7.7% (151)	13.5% (267)
	Saguenay campus and others	9.2% (181)	21.7% (427)
Household	Alone	18.6% (389)	19.3% (380)
	Family (with/without children)	34.3% (717)	41.2% (810)
	Co-renters or others	40.8% (853)	34.2% (673)
	Parents' or family's home	6.4% (133)	5.3% (103)
Home ownership status	Tenants	81.9% (1717)	74.3% (1465)
Annual family income (CAD)	Less than $15,000	41.3% (812)	34.1% (672)
	$15,000 to $24,999	17.3% (340)	16.1% (316)
	$25,000 to $54,999	13.7% (269)	15.4% (304)
	$55,000 and more	20.3% (399)	27.6% (544)
	Refusal of unknown	7.5% (148)	6.7% (133)

* Weighted for sex, group age and campus affiliation.

### 3.2. Global Prevalence and Geographic Distribution

More than a third (36.0%, 95% CI: 33.9–38.1) of surveyed students reported exposure to at least one sign of residential dampness or mold. In descending order, the most reported signs were: excessive humidity (21.4%), visible mold (16.6%), history of water leaks (9.8%) and finally mold odor (7.4%). Due to variation coefficients that were superior to 15%, the geographic analysis of residential dampness or mold prevalence was not possible for each investigated Quebec region or Sherbrooke district. Nevertheless, residential dampness or mold prevalence in student housing was 37.4% (95% CI: 34.7–40.1) for the Eastern Townships, which was similar to Montreal (39.7%, 95% CI: 32.4–46.9), but significantly higher than the neighboring Montérégie region (29.7%, 95% CI: 23.2–36.2). When looking more closely at Sherbrooke districts, Mont-Bellevue, where the main campus is located, had the highest proportion of residential dampness or mold (41.0%, 95% CI: 37.3–44.7). Mont-Bellevue’s prevalence was statistically higher than the overall prevalence as well as that of other Sherbrooke districts. For comparison purposes, the Fleurimont (health campus) and Jacques-Cartier districts had a prevalence of 32.0% (95% CI: 25.7–38.4) and 31.1% (95% CI: 24.3–37.9), respectively.

### 3.3. Socio-Demographic Risk Factors

The reported prevalence of residential dampness or mold was examined according to various socio-demographic characteristics (Table 2).

**Table 2:** Prevalence of residential dampness or mold in student housing by socio-demographic characteristics, Université de Sherbrooke students (QC, Canada), winter 2014.

Characteristic	Prevalence % (n)	*p* Value (χ^2^ Test)	Odd Ratio (95%CI)
**Sex**			
Men	35.5% (306)		1
Women	36.4% (404)	0.694	1.04 (0.86–1.25)
**Age**			
18 to 23 years old	36.2% (286)		1
24 to 30 years old	37.3% (214)		1.05 (0.84–1.31)
31 years old and more	34.6% (211)	0.623	0.93 (0.75–1.16)
**Birth country**			
Canada	35.6% (593)		1
Other countries	38.6% (117)	0.310	1.14 (0.89–1.47)
**Spoken language at home**			
French spoken at home	36.1% (688)		1
French not spoken at home	34.3% (23)	0.762	0.92 (0.55–1.54)
**Mode of study**			
Full time	36.9% (574)		1
Part time	33.2% (136)	0.163	0.85 (0.67–1.07)
**Grade**			
Baccalaureates	37.4% (380)		1
Masters	36.7% (257)		0.97 (0.80–1.19)
Doctorates or post-doctorates	29.0% (72)	0.045	0.69 (0.51–0.93)
**Annual family income (CAD)**			
Less than $15,000	39.7% (267)		1
$15,000 to $24,999	42.0% (133)		1.10 (0.84–1.44)
$25,000 to $54,999	36.5% (111)		0.87 (0.66–1.15)
$55,000 or more	29.4% (160)		0.63 (0.50–0.80)
Refusal or unknown	27.8% (37)	<0.001	0.58 (0.38–0.87)
**Number of residents in housing**			
1 to 2	33.2% (379)		1
3 to 4	41.1% (270)		1.40 (1.15–1.71)
5 and more	36.2% (47)		1.14 (0.78–1.67)
Unknown or not understood	31.8% (14)	0.009	0.94 (0.49–1.79)
**Household composition**			
Alone	29.5% (112)		1
Family (with/without children)	36.3% (294)		1.36 (1.04–1.77)
Co-renters or others	41.4% (279)		1.69 (1.29–2.21)
Parents’ or family’s home	23.3% (24)	<0.001	0.73 (0.44–1.21)
**Home ownership**			
Tenants	39.7% (581)		1
Owners	25.5% (129)	<0.001	0.52 (0.42–0.65)
**Previous insalubrious housing experience **			
No experience	35.0% (631)		1
Have left an insalubrious residence	46.5% (79)	0.003	1.61 (1.17–2.21)

Most factors (sex, age, country of birth, language spoken at home and mode of study) were not associated with a reported exposure to residential dampness or mold. However, annual family income was inversely associated with dampness or mold. Indeed, students with an annual family income of more than CAD55,000 were less likely to declare such problems when compared with those earning less than CAD15,000 (OR = 0.63, 95% CI: 0.50–0.80). Doctorate and post-doctorate students, who generally had a higher family income (unpublished data: *p* < 0.001), were also less likely to report signs of dampness or mold (OR = 0.69, 95% CI: 0.51–0.93) when compared to baccalaureate students. Residential dampness or mold was more frequently reported by tenants than owners (OR = 1.92, 95% CI: 1.54–2.38). Students sharing an apartment with two or three other residents were more likely to report signs of residential dampness or mold than students living alone or with only one person (OR = 1.40, 95% CI: 1.15–1.71). When looking more closely at household composition, students living with co-tenants and those in a family unit (with or without children) declared significantly more dampness or mold problems compared to students living alone, while students living in their parents’ home did not. Interestingly, students who reported leaving an insalubrious residence in the past were still more prone to stay in such homes (OR = 1.61, 95% CI: 1.17–2.21).

### 3.4. Housing Risk Factors

As described in Table 3, reported exposure to residential dampness or mold is significantly associated with buildings which: (1) had 2 to 11 apartments; (2) were of older construction; (3) needed renovations and (4) were not occupied by the landlord (all *p* < 0.001). It is noteworthy that these building characteristics were very frequent among the surveyed students. The need for housing renovations was the strongest factor associated with residential dampness or mold.

**Table 3:** Prevalence of residential dampness or mold per housing characteristics, Université de Sherbrooke students (QC, Canada), winter 2014.

Housing Characteristic	Prevalence % (n)		*p* Value (χ^2^ Test)	Odd Ratio (95%CI)
**Type of residence**				
One apartment only	32.6% (202)			1
2 to 11 apartments	41.6% (331)			1.48 (1.19–1.84)
12 apartments or more	34.4% (147)			1.09 (0.84–1.41)
University-owned accommodations	24.8% (27)		<0.001	0.67 (0.42–1.08)
**Year of construction**				
Before 1961	44.5% (146)			1
1961–1980	45.7% (269)			1.05 (0.80–1.38)
1981–2000	34.2% (156)			0.65 (0.49–0.87)
After 2000	17.9% (64)			0.27 (0.19–0.39)
Unknown	31.1% (75)		<0.001	0.56 (0.40–0.80)
**Monthly rental costs (tenants only, CAD)**				
$0 to $499	36.6% (146)			1
$500 to $999	40.9% (361)			1.20 (0.94–1.53)
$1000 and more	43.4% (59)		0.239	1.32 (0.89–1.96)
**Monthly property costs (home owners only, CAD)**				
$0 to 999	29.8% (67)			1
$1000 and more	23.9% (49)		0.170	0.74 (0.48–1.14)
**Need for housing renovations**				
Regular maintenance	22.3% (269)			1
Minor needs	49.4% (269)			3.40 (2.74–4.22)
Major needs	77.8% (172)		<0.001	14.78 (9.99–21.88)
**Owner proximity**				
Out of building	40.5% (495)			1
In building, but out of the apartment	35.7% (86)			0.81 (0.61–1.08)
In the apartment	25.3% (129)		<0.001	0.50 (0.40–0.63)
**Heating system**				
Electrical heaters	36.2% (508)			1
Hot water radiators	37.3% (94)			1.05 (0.80–1.39)
Central system	33.3% (47)			0.89 (0.62–1.29)
Others	39.6% (44)			1.17 (0.79–1.73)
Unknown heating system	25.4% (16)		0.360	0.61 (0.34–1.09)
**Presence or absence of some characteristics:**				
**Bedroom in basement**	No	34.3% (557)		
	Yes	44.5% (154)	<0.001	1.53 (1.21–1.93)
**Carpeting**	No	34.6% (591)		
	Yes	44.9% (119)	0.001	1.54 (1.18–2.00)
**Rodents**	No	35.1% (651)		
	Yes	51.7% (60)	<0.001	1.98 (1.36–2.89)
**Dehumidifier**	No	32.6% (523)		
	Yes	51.2% (187)	<0.001	2.18 (1.73–2.74)
**Humidifier**	No	35.9% (666)		
	Yes	38.3% (44)	0.606	1.11 (0.75–1.63)
**Thermostat**	No	37.7% (89)		
	Yes	35.8% (621)	0.564	0.92 (0.70–1.22)
**Air conditioning**	No	37.9% (530)		
	Yes	31.5% (180)	0.008	0.76 (0.61–0.93)
**Central mechanical ventilation**	No	41.8% (499)		
	Yes	27.1% (211)	<0.001	0.52 (0.43–0.63)
**Cooker hood connected to exterior**	No	44.0% (270)		
	Yes	32.4% (440)	0.004	0.61 (0.44–0.85)
**Bathroom ventilation**	No	44.8% (69)		
	Yes	33.2% (533)	<0.001	0.61 (0.50–0.74)

Respondents living in buildings in need of minor and important renovations were significantly more at risk (OR = 3.40, 95% CI: 2.74–4.22 and OR = 14.78, 95% CI: 9.99–21.88, respectively) than those living in housing only requiring regular maintenance. Residential dampness or mold was not found to be associated with either monthly property expenses (home owners) or monthly rental costs (tenants). The type of heating system and the presence of a thermostat were not associated with residential dampness or mold, but many other housing characteristics were. These factors included the presence of a bedroom in the basement, of carpeting, of rodents, and of a dehumidifier. On the other hand, a variety of ventilation systems (air conditioning, central mechanical exhaust, cooker hood and bathroom exhaust) were all significantly associated with less frequent residential exposure to dampness or mold (*p <* 0.01). Among ventilation components, the presence of a central mechanical ventilation system had the strongest negative association with dampness or mold. Indeed, students having this type of system had an almost two-fold decrease in the likelihood of declaring residential dampness or mold signs (OR = 0.52, 95% CI: 0.43–0.63).

A validation analysis using “mold odor” instead of “any dampness or mold sign” as exposure was conducted given that mold odor is a likely sign of insufficient ventilation but also a greater problematic indicator [24]. Even though exposure to mold odor was less frequent (reported by 7.4% of students), its associations with many students and housing characteristics (e.g., tenant status, number of occupants in housing, housing in a basement, older building, past experience of living in insalubrious housing and lack of ventilation systems) was stronger.

## 4. Discussion

Our findings show an overall self-reported prevalence of residential dampness or mold of 36.0% in this study population of university students. This is consistent with results from a previous study in a US college student population which reported that 39.3% of students’ housings had signs of mold on surfaces [14]. This prevalence is also very similar to the proportion of reported dampness by the general population in a study conducted in six Canadian regions (37.6%) [43] and in a recent study of Montreal households which included basement housing in its exposure criteria (36.3%) [22].

Even though these similarities in prevalence could suggest no increased risk in mold exposure for university students, additional analyses showed otherwise. Indeed, when considering only tenant students (which represent over 81.9% of the study sample), the observed dampness or mold prevalence of 39.7% was closer to the general population prevalence estimated in some problematic Montreal districts [22]. Geographically, this study also confirmed that students living in the main university campus district of Mont-Bellevue were affected by residential dampness or mold at a greater extent (41.0%, 95% CI: 37.3–44.7) than those living elsewhere. This finding was expected since a high building renovation needs in this district was identified by a recent Canadian census [15]. Furthermore, our findings are higher than a previous exposure prevalence of 15.0% observed in a Finnish student population [21]. This is coherent with another European study demonstrating that temperate climates (such as in Quebec’s southern regions) present a higher prevalence of residential mold, water damage or a combination of both, than colder climates (such as in Finland) [21].

The study findings support the initial assumption that students are vulnerable to residential dampness or mold problems and further emphasize the high variability of exposure according to socio-demographics and housing characteristics. This is important given that university students are often tenants, often have a low income and often live in older multi-unit apartment buildings, all factors which were associated with exposure to residential dampness or mold. Such characteristics have also been found to be associated with dampness or mold in previous studies with similar or even stronger associations [13,17]. For example, our study showed that tenant-occupied housings were about twice more likely (OR = 1.92, 95% CI: 1.54–2.38) to present signs of dampness compared to owner-occupied buildings. A Swedish survey conducted in the general population between 1998 and 2000 estimated this increased likelihood to be of the order of 5.47 (95% CI: 3.03–9.87) [13]. On the other hand, in our study, student housing costs were not associated with residential dampness or mold. This absence of association was also reported in a similar study on U.S. students [14]. However, our study added that family income was inversely associated with exposure to dampness or mold. One explanation could be that higher family income meant higher empowerment for housing selection at a better price and higher maintenance skills. Low family income could also play a proxy role for housing vulnerability as it has also been associated with many vulnerability factors such as ownership status, building type, its year of construction, the need for renovations and the type of ventilation (results not shown). The association between students reporting past experiences of living in damp or moldy housing and present residential exposure to dampness or mold corroborates this vicious circle and the lack of empowerment of some students.

Even though some socio-geographic characteristics were associated with residential dampness or mold, our findings highlight housing characteristics as main associated factors. These housing factors were mostly linked to insufficient ventilation (bathroom fan, cooker hood, central ventilation and air conditioning) and lack of maintenance (renovation needs, year of construction, presence of rodents and of a dehumidifier) as described in the literature [1,13,23]. Interestingly, a pooled analysis [44] in the USA has shown that some housing factors, including rodents and below average housekeeping, were associated with higher allergen levels. Similarly to Sharpe *et al.*, who used mold odor as a combination of mold exposure and lack of ventilation [24], our validation analysis demonstrated stronger associations between mold odor (as opposed to any sign of dampness or mold) and many students and housing characteristics. However, previous associations between central heating systems and residential dampness or mold [23] could not be replicated in this study (*p* = 0.360). Considering that electrical and hot water heating systems are usually both visible in housings and that only few students did not know their type of heating system, a classification bias probably does not explain this result. It is also worth mentioning that some students may alter their behavior in terms of heating or ventilation due to concerns with their personal global warming footprint. However, because Quebec’s nationalized electricity production is mostly powered by hydroelectricity, the use of ventilation or heating to reduce mold may have less impact on global warming in Quebec than in other countries using other types of electricity production schemes (e.g., natural gas).

As underlined in a recent provincial public health report [45], access to affordable salubrious housing is a key health determinant, particularly for low socioeconomic populations. In addition to the negative health effects of the housing itself, these housings are often located in generally low socioeconomic neighborhoods which offer fewer services and are closer to sources of pollution (e.g., highways or industrial sectors), which further increases the health inequality gap felt by individuals with a low socioeconomic status. Populations with a lower socioeconomic status are not only more at risk to be exposed to residential dampness or mold, but they also are likely to have less knowledge, fewer skills and limited access to resources. We demonstrated that many university students, who are usually not considered a vulnerable population, are frequently exposed to residential dampness or mold and may have challenges in moving away from their insalubrious environment. A combination of proximity and socioeconomic difficulties leads to concentrated low-cost student housing in neighborhoods near universities, like the Mont-Bellevue district of Sherbrooke. This situation may favor a lack of building maintenance in those neighborhoods (thus “high building renovation needs”), which could lead to a higher prevalence of residential dampness or mold.

By its large and geographically-spread out sample composed of a rarely studied population, this unique study successfully highlights the usefulness of targeting vulnerable populations using various student and housing characteristics, such as: year of construction, ownership status and maintenance needs. This latter key variable is usually collected in the national census [15] and therefore may be very useful for public health and city officials to identify high-risk neighborhoods. Considering that housing investments have been associated with health improvements [25], strategies to invest in residential ventilation systems and in preventive building maintenance could lead to better global air quality [1] and may help to prevent health issues related to residential dampness or mold.

### Limitations

The low response rate encountered in this study may have led to non-response bias. Therefore, exposed students may have self-selected in greater proportions than unexposed ones, leading to the over-estimation of mold exposure. Similarly, students having adverse characteristics, such as low socioeconomic status, may have been over-represented. To correct for younger students, women and students affiliated to the main campus responding more frequently to the survey, results were weighted for key socio-demographic characteristics of the study population. Furthermore, comparable results were reported in previous studies based on similar populations and with higher response rates [14,22]. Despite these corrections and comparisons, the study results should nevertheless be interpreted with caution given the low participation rate of 8.1%.

When focusing on data collection, dampness or mold signs were self-reported and not objectively measured. Therefore, results could have also suffered from a classification bias, and could have been under- or over-reported. However, studies have shown that occupant reports represent a valid estimation of dampness or mold exposure (sensitivity of 75% and specificity 71% when using the hygienist evaluation as a gold standard) [34]. This estimation, although generally underestimated, is even closer to biological measures than a visual evaluation by an expert [1,34,46]. As students were independently surveyed, more than one co-tenant per residence could have been included in the study. To protect their confidentiality, the complete residential address of respondents was not collected and it was therefore impossible to eliminate these possible duplicates. Moreover, the fact that some students may be temporarily travelling for internship purposes or going back to their families during holidays may limit their exposure to university-term housing. Because time spent away from term-housing was not assessed in this study, we may have overestimated the prevalence of residential exposure to dampness or mold.

Finally, this is a cross-sectional study which cannot evaluate temporality or causal links. Such a limitation could have given rise to associations such as the one between the presence of a dehumidifier and the presence of mold, the former being probably a consequence rather than a causal factor of the latter. However, the main purpose of the current study was to describe the distribution of residential mold problems according to socio-geographic and housing factors and not to identify predictors of such problems. This study therefore offers a valuable description of student housing conditions and associated factors for residential dampness or mold that could help stakeholders identify student populations at increased risk for such exposure and better target their interventions.

## 5. Conclusions

Adding to a growing body of knowledge, these results suggest the need for public health authorities, university administrators and city officials to regard university students as a vulnerable population at risk of living in damp or moldy housings. These results especially emphasize the variability of this risk amongst university students which are a heterogeneous population. Indeed, despite the fact that many of them share at risk characteristics such as low income and tenant status, others are much less at risk to be exposed. Thus, targeting all university students with interventions could be inefficient compared to focused interventions for those most at risk. Despite not having examined intervention efficacy and considering the limits of this study, these results underline which students’ accommodations are more at risk to be associated to a damp or moldy environment and could be targeted for focused interventions. However, further research is needed to fully evaluate the health and academic consequences of residential dampness or mold in university students. This research, which should have strong study designs, may ultimately lead to targeted environmental interventions directed (1) on knowledge transfer to student populations about environmental health issues and their right as tenants and (2) on reinforcing housing regulations (incentives or tax penalties) in order to enhance the landlord’s responsibility with regards to salubrious housing. These studies should also help public health professionals to better understand and protect university students from such insalubrious housing.
